# The medaka *dhc2* mutant reveals conserved and distinct mechanisms of Hedgehog signaling in teleosts

**DOI:** 10.1186/s12861-015-0057-x

**Published:** 2015-02-03

**Authors:** Takayoshi Yamamoto, Tatsuya Tsukahara, Tadashi Ishiguro, Haruo Hagiwara, Masanori Taira, Hiroyuki Takeda

**Affiliations:** Department of Biological Sciences, Graduate School of Science, University of Tokyo, 7-3-1 Hongo, Bunkyo, Tokyo, 113-0033 Japan; Department of Anatomy and Cell Biology, Teikyo University School of Medicine, 2-11-1 Kaga, Itabashi, Tokyo, 173-8605 Japan; Present address: Institute of Molecular and Cellular Biosciences, University of Tokyo, 1-1-1 Yayoi, Tokyo, 113-0032 Japan

**Keywords:** Hedgehog signaling, Cilia, Medaka fish, Fused, dhc2, Cytoplasmic dynein heavy chain 2, Neural tube

## Abstract

**Background:**

Primary cilia are essential for Hedgehog (Hh) signal transduction in vertebrates. Although the core components of the Hh pathway are highly conserved, the dependency on cilia in Hh signaling is considered to be lower in fish than in mice, suggesting the presence of species-specific mechanisms for Hh signal transduction.

**Results:**

To precisely understand the role of cilia in Hh signaling in fish and explore the evolution of Hh signaling, we have generated a maternal-zygotic medaka (*Oryzias latipes*) mutant that lacks *cytoplasmic dynein heavy chain 2* (*dhc2*; MZ*dhc2*), a component required for retrograde intraflagellar transport. We found that MZ*dhc2* exhibited the shortened cilia and partial defects in Hh signaling, although the Hh defects were milder than zebrafish mutants which completely lack cilia. This result suggests that Hh activity in fish depends on the length of cilium. However, the activity of Hh signaling in MZ*dhc2* appeared to be higher than that in mouse *Dnchc2* mutants, suggesting a lower requirement for cilia in Hh signaling in fish. We confirmed that Ptch1 receptor is exclusively localized on the cilium in fish as in mammals. Subsequent analyses revealed that Fused, an essential mediator for Hh signaling in *Drosophila* and fish but not in mammals, augments the activity of Hh signaling in fish as a transcriptional target of Hh signaling.

**Conclusions:**

Ciliary requirement for Hh signaling in fish is lower than that in mammals, possibly due to *fused*-mediated positive feedback in Hh signaling. The finding of this fish-specific augmentation provides a novel insight into the evolution of Hh signaling.

**Electronic supplementary material:**

The online version of this article (doi:10.1186/s12861-015-0057-x) contains supplementary material, which is available to authorized users.

## Background

Hedgehog (Hh) signaling is an evolutionarily conserved signal transduction pathway which is essential for various aspects of embryogenesis including patterning events of the vertebrate neural tube and limb [1,2]. The mechanism of Hh-signal transduction has been the target of intense studies but remains only partially understood. One of the striking features of Hh signaling is that the primary cilium, a microtubule-based, immotile cellular protrusion, is essential for Hh signaling in vertebrates but not in *Drosophila* [3]. A requirement for the cilium in this pathway was first identified by genetic screening in mice for ciliary mutants exhibiting phenotypes similar to those of Hh-pathway mutants [4]. However, subsequent genetic and molecular analyses demonstrated that cilium-dependency and the mediators of Hh signaling varies between fish and mammals, raising a question about conservation and evolution of the mechanism of Hh-signal transduction [5].

The formation and maintenance of cilia depend on the conserved process of intraflagellar transport (IFT) [6]. Ciliary proteins are transported along the ciliary axoneme by IFT machinery, driven by kinesin-based anterograde and dynein-powered retrograde transport. In the absence of Ift88, a component of anterograde IFT machinery, both mouse and zebrafish embryos lack all cilia and exhibit a severe reduction in Hh signaling. However, the phenotype is milder in zebrafish. In the neural tube, most of the Hh target genes are not expressed in mouse mutants, while the expression of low-threshold genes remains and expands in zebrafish [4,5], suggested that cilium is required for Hh signaling also in fish, but the dependency on cilia is lower than that in mammals. However, it was still unclear how much Hh signaling in fish depends on cilia and what is the underlying mechanism for that difference.

Furthermore, Fused (Fu), a putative serine-threonine kinase, first identified as an essential mediator of Hh signaling in *Drosophila*, turned out to be dispensable for mammals, but it is indispensable for zebrafish [7-10]. These facts suggest that the pathway in zebrafish is more similar to that in *Drosophila* or placed in between *Drosophila* and mammals, making fish a unique model with which to investigate the transition state from ancestral to modern type of Hh signaling.

To further address this, we have generated a maternal-zygotic medaka mutant that lacks *cytoplasmic dynein heavy chain 2* (*dhc2*; MZ*dhc2*), an essential component of retrograde IFT, and compared the neural phenotypes of medaka and mouse mutants (*Dnchc2*). We confirmed that the requirement for cilia in Hh signaling is lower in fish and revealed that the Hh activity in fish depends on the length of cilium. Additionally, Ptch1 receptor is localized to cilia in fish as in mammals. Subsequent analyses revealed that the difference in the requirement for cilia in Hh signaling across vertebrates can be interpreted by differential regulation and function of Fu.

## Results

### Generation of maternal-zygotic *aA90*/*dhc2* mutants

**Figure 1:**
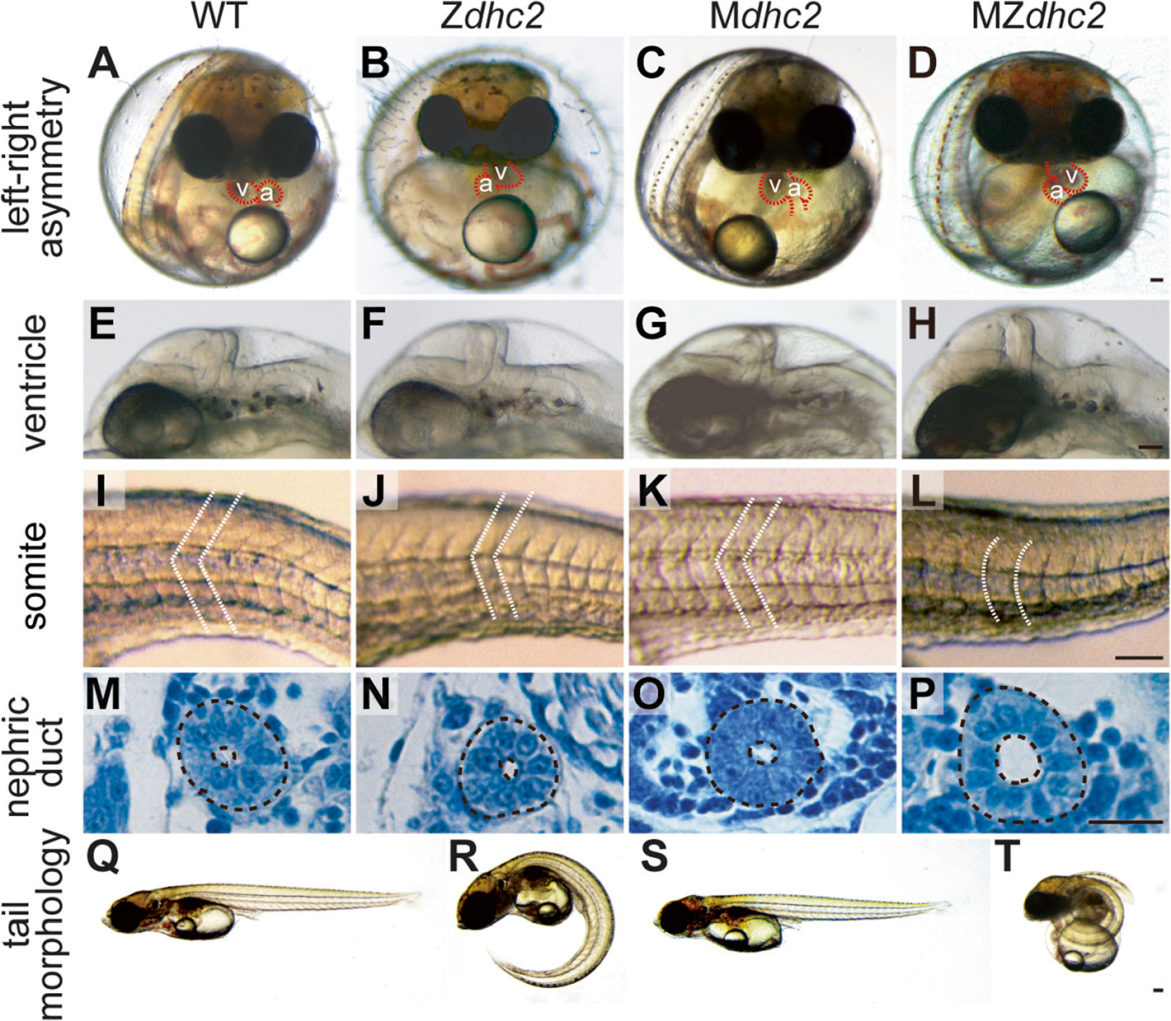
**Morphological phenotypes of** ***dhc2*** **mutants. (A-D)** Frontal views of the heart at 6 days post fertilization (dpf). **(E-L)** Lateral views of the ventricle **(E-H)** and the somite **(I-L)** at 3 dpf. **(M-T)** Transverse section of nephric duct **(M-P)** and tail morphology at 7 dpf **(Q-T)**. v, ventricle; a, atrium; Scale bars: 100 μm in **D**, **H**, **L**, **P**; 200 μm in **T**.

The medaka *aA90* mutant, isolated in an ENU-induced mutagenesis screening [11], is a recessive lethal mutant showing defects in left-right (L/R) axis determination (Figure 1A-B). L/R asymmetry is established by directional flow of extra-embryonic fluid surrounding the node (Kupffer’s vesicle in fish) by cilia. To identify the defective gene in *aA90* mutant, we carried out positional cloning and narrowed down the *aA90* locus to a 250 kb region in linkage group 13, which harbors a single predicted open reading frame, *cytoplasmic dynein heavy chain 2* (*dhc2*), an IFT retrograde component (Additional file 1: Figure S1A). We found that *aA90* has a 37.7 kb deletion in the *dhc2* locus including the start codon, the heavy chain (HC)-HC, the HC-Intermediate chain interaction domain, and the AAA ATPase domain (Additional file 1: Figure S1A, C). Database searches demonstrated that the *dhc2* gene exists as a single copy within the medaka genome. Due to long and complicated structure of the *dhc2* gene (expands over 176 kb with 98 exons), we were unable to perform a rescue experiment by RNA injection. Injection of antisense morpholinos (MO) against the *dhc2* gene into wild-type embryos significantly phenocopied *aA90*, which led us to conclude that *dhc2* is the gene deficient in the *aA90* mutant (Table 1).

**Table 1:** **Defects in heart asymmetry in** ***dhc2*** **mutant embryos and morphants**

**Genotype**	**n**	**Correct (%)**	**Reversed (%)**
Wild type	110	99.1	0.9
Z*dhc2*	294	76.2	23.8
*dhc2* MO*	108	77.8	22.2
M*dhc2*	82	100	0
MZ*dhc2*	128	46.9	53.1

*dhc2-Met MO, 5′-AAATGCGGCAGACTCGCAGTTTTAC-3′.

**Figure 2:**
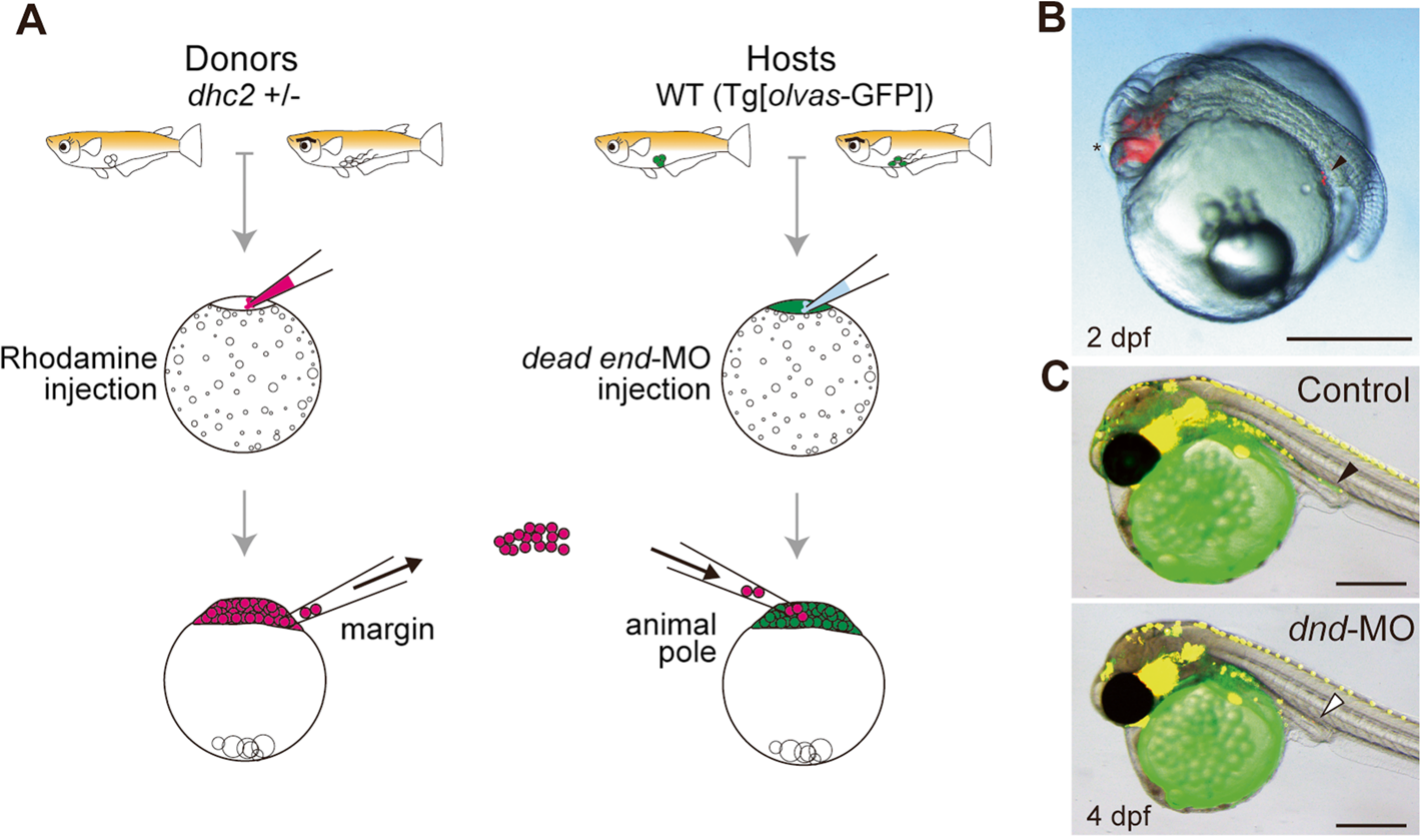
**Generation of Maternal-Zygotic** ***dhc2*** **mutant.** Germ-line replacement strategy using the rhodamine-dextran labeling technique. **(A)** Overview of transplantation strategy showing the transfer of cells from the margin of rhodamine-dextran-labeled mutant donor embryos into the animal pole of *dead end*-MO injected WT (Tg[*olvas*-GFP], germ cells are labeled with GFP [29]) hosts. A morpholino antisense oligonucleotide (Genetools) to *dead end* was complementary to a region covering the splicing site for exon 2 and intron 2, 5′-TGTTCAGAACTGGCCTCTCACCATC-3′. **(B)** Chimeric host embryos were screened at 2 dpf for the presence of rhodamine-labeled donor PGCs that had migrated successfully into the gonadal mesoderm (arrowhead). Host embryos also showed somatic contribution of rhodamine-dextran-labeled donor cells to anterior neuroectoderm lineages (*). **(C)** Chimeric host embryos were screened again at 4–6 dpf for the lost of GFP-labeled host PGCs at the dorsal region of the gut (arrowhead). Scale bars: 500 μm in **B**-**C**.

Probably due to the maternal contribution of *dhc2*-gene products, the phenotype of *aA90*/*dhc2* mutants was mild. For example, only one-fourth of the *aA90*/*dhc2* homozygous mutants showed *situs inversus* (Table 1). To completely eliminate *dhc2* products, we have generated maternal-zygotic *aA90*/*dhc2* mutants using the germline-replacement technique [12,13] with some modifications (Figure 2). Crosses of females with mutant germ cells and heterozygous males (*dhc2*/+) generated 50% homozygous mutants that lacked both maternal and zygotic products of *dhc2* (MZ*dhc2*) and 50% heterozygous mutant embryos that lacked only the maternal *dhc2* contribution (M*dhc2*).

**Figure 3:**
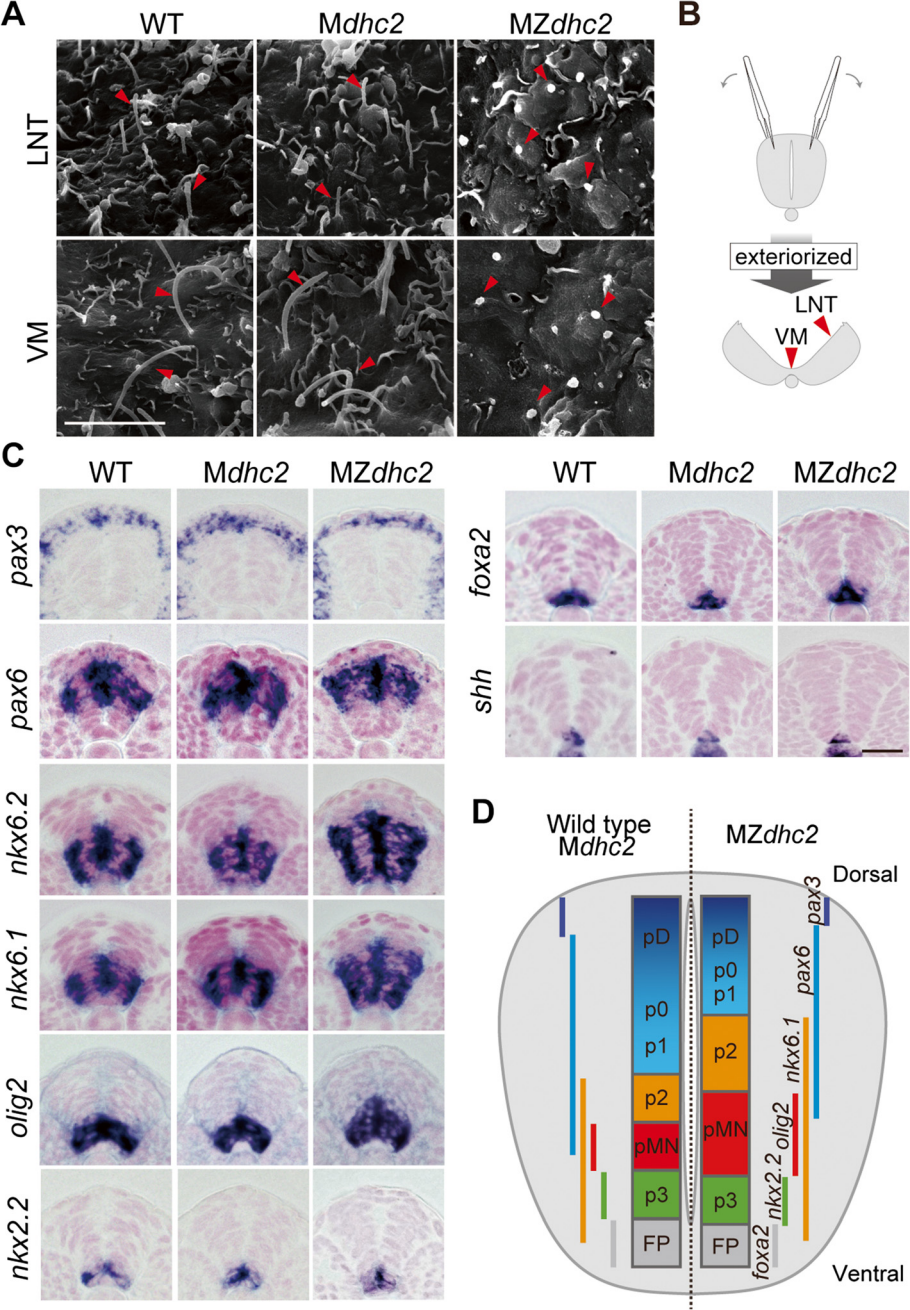
**Cilia and Neural patterning in MZ** ***dhc2*** **mutants. (A)** SEM analysis of the ventricular surface of the neural tube at 16-somite stage. **(B)** Schematic view of opening of the apical surface of neural tube with forceps. **(C)** Expression of neural tube markers in a cross-sectional view at 16-somite stage (Dashed line in Additional file 3: Figure S3 indicates section plane). **(D)** Representation of the size of each progenitor domain along the DV axis. Scale bars: 5 μm in **A**; 20 μm in **C**.

As expected, the complete loss of *dhc2* activity increased the frequency of *situs inversus* to 52.8% (Table 1, Figure 1A-D) as well as enlarged ventricles and expanded nephric duct (Figure 1E-H, M-P). Moreover, the typical phenotypes of defective Hh-signaling, severe ventral curvature and U-shaped somites instead of chevron-shaped ones, were observed in MZ*dhc2* mutants, but not in zygotic (Z*dhc2*) or M*dhc2* mutants (Figure 1I-L, Q-T), indicating reduced levels of Hh signaling. Importantly, the morphology of cilia was dramatically shortened as demonstrated by scanning electron microscopy (SEM) (Figure 3A). To expose the ventricular surface area of neural tubes, we exteriorized this area with forceps, prior to fixation (Figure 3B) and found that cilia on the surface of non-floor plate (FP) neuroepithelial cells (LNT, lateral neural tube) and longer ones on the FP cells (VM, ventral midline) were much shorter and bloated in MZ*dhc2* than their wild-type counter parts (Figure 3A). In the Kupffer’s vesicle and somites, cilia were shortened in MZ*dhc2*, as compared with those in WT, M*dhc2* and Z*dhc2*. The ciliary morphology in Z*dhc2* mutants appeared normal at least until the segmentation stages, but subtle defects in function or lately overt defects could account for their milder phenotypes (Table 1, Additional file 2: Figure S2, data not shown). The ciliary phenotypes in MZ*dhc2* mutants are nearly identical to those in mouse *Dnchc2* −/− [14,15], and thus the analysis of the Hh activity in MZ*dhc2* mutants enabled us to examine differences and distinct mechanisms between fish and mouse in the requirement for cilia in Hh signaling.

### Patterning of the spinal cord in MZ*dhc2* mutants

In the neural tube, sonic hedgehog (Shh) ligand forms a dorso-ventral (DV) gradient with the highest concentration ventrally, and specifies cell fates in a concentration-dependent manner [16]. Thus, the expression of cell-type specific genes serves as a readout of Hh activity and delineates domains in the ventral neural tube. Roughly, from ventral to dorsal, gene expression is as follows: *foxa2* in the FP, *nkx2.2* in p3 neuron precursors, *olig2* in motor neuron precursors (pMN), *nkx6.1*/*6.2* in p3/pMN/p2 progenitors, and *pax6*, *pax3*, *dbx1* and *dbx2* in dorsally located neuron precursors and their expressions are mutually exclusive underlined by their repressive interactions (Figure 3D) [17,18]. Shh is known to induce the expression of the ventral genes (*foxa2*, *nkx2.2*, *olig2*, *nkx6.1* and *nkx6.2*), while suppressing the dorsal genes (*pax6*, *pax3*, *dbx1* and *dbx2*) [17-19]. We first confirmed that *shh* was normally expressed in the medial FP (MFP) and underlying notochord of MZ*dhc2* mutants (Figure 3C, Additional file 3: Figure S3J), suggesting that defects observed in mutants are mainly ascribed to signal transduction defects.

In MZ*dhc2* mutants, *foxa2* and *nkx2.2* were expressed (Figure 3C, Additional file 3: Figure S3H-I), whereas ventral intermediate genes, *olig2*, *nkx6.1* and *nkx6.2* were dorsally expanded and this dorsal expansion was not observed in Z*dhc2* (Figure 3C, Additional file 3: Figure S3E-G). Dorsal expansion of *olig2* expression in MZ*dhc2* was also observed at three different axis levels (Additional file 4: Figure S4). The expression of these ventral genes suggests that the Hh pathway is activated in cells with severely shortened cilia and even reaches the high levels of activation on the most ventral side. Like zebrafish, *foxa2* expression in the medial FP is Hh-independent in medaka embryos (Additional file 5: Figure S5B), and thus we will use *nkx2.2* expression as a marker of the high level of Hh activation. Also, it is worth noting that the most ventral region appeared to be missing in MZ*dhc2* embryos, as the expression domains of *nkx2.2*, separated by the negative medial FP cells, frequently merged in the medial region (Figure 3C). However, due to the lack of a specific marker for this region, we were unable to determine a cell type specifically defective in MZ*dhc2* embryos.

**Figure 4:**
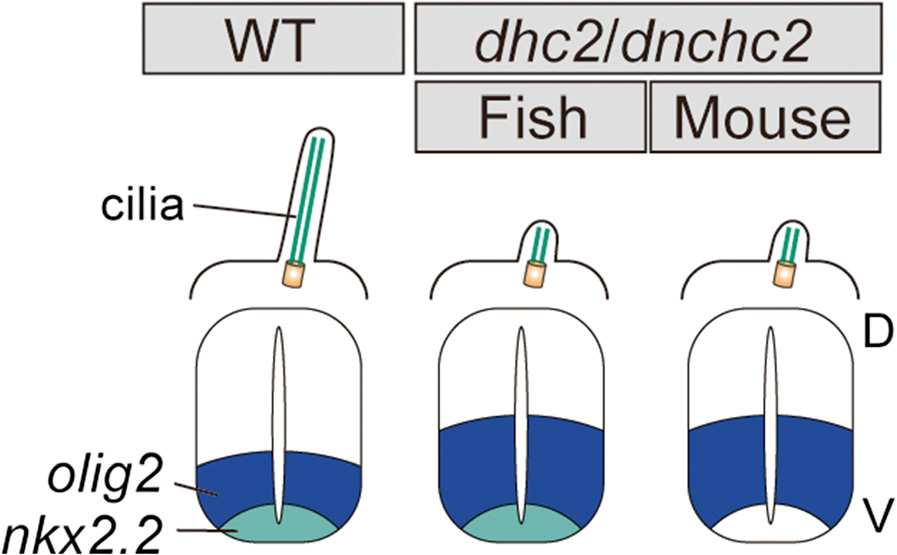
**A schematic drawing explaining the similarities and differences in ciliary and neural tube phenotypes between fish and mouse** ***dhc2*** **/** ***dnchc2*** **mutants.** Ciliary phenotypes and dorsal expansion of *olig2* domain in MZ*dhc2* mutants are nearly identical to those in mouse mutant but *nkx2.2* expression was reported to be lost in mouse mutant [14,15]. D, dorsal; V, ventral.

The expansion of lower-threshold gene expression (*olig2*, *nkx6.1* and *nkx6.2*) also suggests that the area of low Hh activation abnormally expanded dorsally in the mutant neural tube. This was further supported by dorsally retracted expression of *pax6*, *pax3*, *dbx1* and *dbx2*, observed in MZ*dhc2* mutants (Figure 3C, Additional file 3: Figure S3A-D). In *Dnchc2*-mutant mice, *nkx2.2* expression was reported to be lost, but *olig2* was expanded [14,15]. Thus, there are similarities and differences in the neural tube phenotypes between fish and mouse *dhc2* mutants (Figure 4), both of which we addressed in the following experiments.

### Lower Hh pathway activation in mutant cells

**Figure 5:**
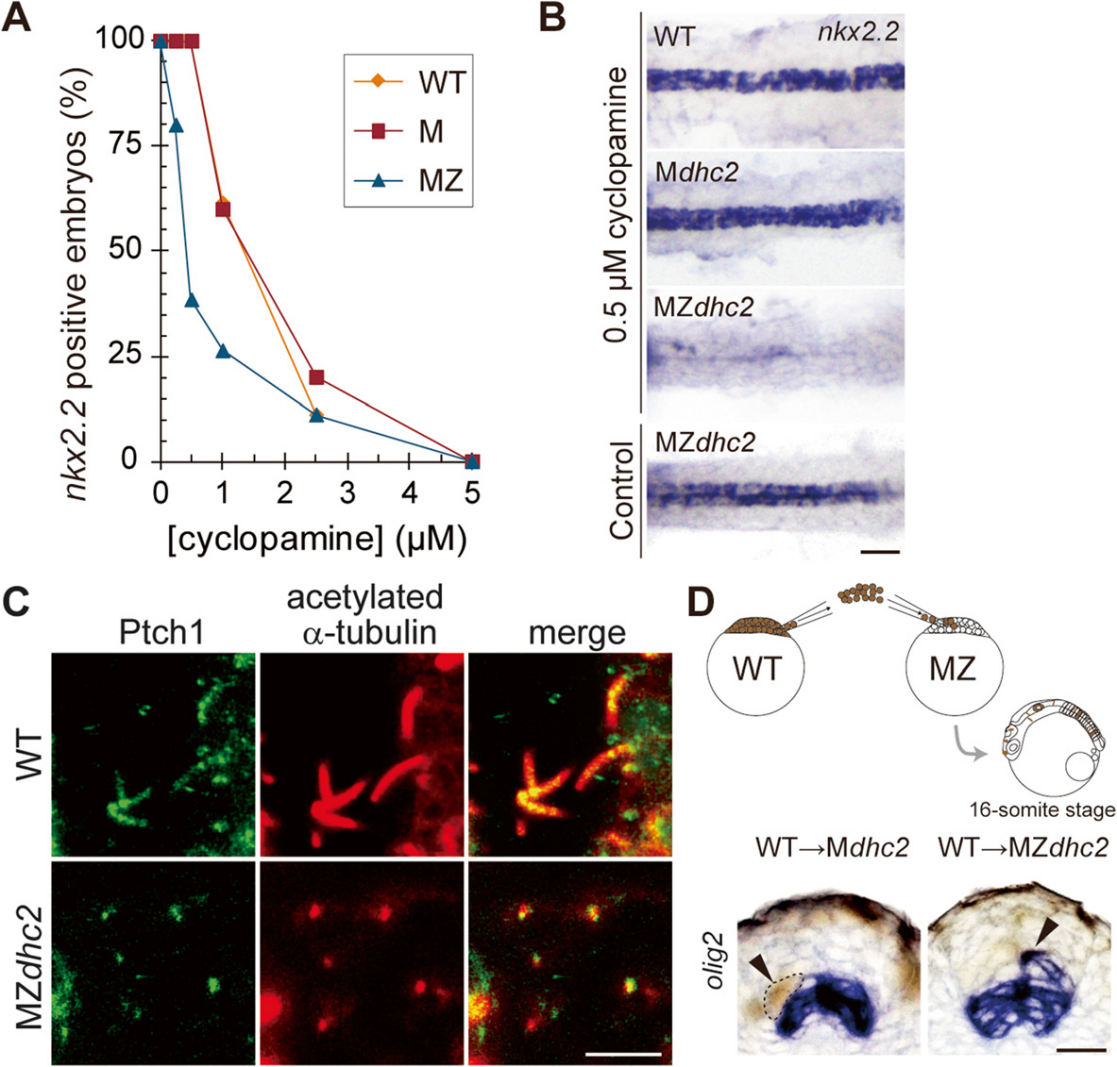
**Hh signaling activity is partially defective in MZ** ***dhc2*** **mutants but Ptch1 is localized to the cilia. (A)** The percentage of *nkx2.2*-positive embryos with the graded series of cyclopamine treatment (Sample numbers are indicated in Additional file 6: Table S3). **(B)** Dorsal view of *nkx2.2* expression in 0.5 μM cyclopamine treated and control (DMSO-treated) embryos. **(C)** Localization of Ptch1 on cilia stained with the anti-acetylated α-tubulin antibody in the neuroepithelium at 16-somite stage. **(D)** Transplantation of biotin-injected WT cells into MZ*dhc2* cells, with its schematic view, resulted in ectopic *olig2* expression of WT cells in the dorsal region of MZ*dhc2* neural tube (arrowhead). Scale bars: 100 μm in **B**, 5 μm in **C**; 20 μm in **D**.

To examine the activation level of Hh pathway in mutant cells, we treated MZ*dhc2* embryos with various concentrations of cyclopamine, a potent antagonist of Smoothened (Smo). Intriguingly, in the MZ*dhc2* group, the percentage of *nkx2.2*-positive embryos started to decrease at a cyclopamine concentration as low as 0.25 μM, and went down below 50% at 0.5 to 1 μM, while at such low concentrations, 100% of embryos maintained *nkx2.2* expression in the wild-type and M*dhc2* groups (Figures 5A-B, Additional file 6: Table S3). These results suggest that the activity of Hh signaling in mutant cells is compromised at the level or upstream of Smo, but still high enough to express the ventral-most marker, *nkx2.2*.

### Patched1 localizes to cilia in medaka fish

In murine cells, Ptch1 receptor was reported to localize the primary cilium at least cultured cells and paraxial mesoderm cells [20,21], whereas it is not the case in *Drosophila* which does not require cilia for the reception of Hh [3]. However, Ptch1 localization was unknown in fish. To address this, we generated an antibody against medaka Ptch1 (Additional file 7: Figure S6A-B, Additional file 8: Table S2), and examined the distribution of Ptch1 in wild-type and MZ*dhc2* neural-tube cells. Firstly, we confirmed that Ptch1 expression in medaka is diminished by cyclopamine treatment (data not shown) and in the morphants, the number of Engrailed-expressing cells in somites was increased (Additional file 7: Figure S6D), similar to zebrafish morphant [10].

As shown in Figure 5C, Ptch1 was localized to the cilia of neuroepithelial cells which are exteriorized with forceps before fixing (Figure 3B) in WT. Importantly, Ptch1 was still localized to severely shortened cilia in MZ*dhc2* (Figure 5C). The specificity of the antibody was confirmed by knockdown and overexpression experiments (Additional file 7: Figures S6C, E). These results indicate that the cilium is the site for Hh receptor Ptch1 localization in medaka.

### MZ*dhc2* cells are less sensitive to Shh

Although the activation level of Hh signaling is still sufficient to induce all target genes in mutant cells, the amount of Ptch1 in severely shortened cilia is likely to be decreased. This could explain the higher sensitivity to cyclopamine in the above experiment (Figure 5A-B). In other words, MZ*dhc2* cells could be less sensitive to Shh. To test this idea, we transplanted wild-type cells into MZ*dhc2* blastula or M*dhc2* (control), and examined *olig2* expression when donor cells were localized in host neural tubes (Figure 5D). We determined the genotype of MZ*dhc2* and M*dhc2* by the eye phenotype at 16-somite stage when transplanted embryos were fixed for the analysis (Additional file 9: Figure S7). Remarkably, *olig2*-positive WT cells were frequently found in the region more dorsal to the host *olig2*-expression domain in MZ*dhc2* embryos (Figure 5D, WT to MZ*dhc2*, arrowhead; n = 9/10), while no such ectopic expression was detected in control transplants (Figure 5D, WT to M*dhc2*, arrowhead; n = 15/15). These results demonstrate that Hh-activation of MZ*dhc2* cells is lower than that in WT cells, even if they are exposed to the same concentration of Hh-ligand.

### Fused forms a positive-feedback loop in fish

**Figure 6:**
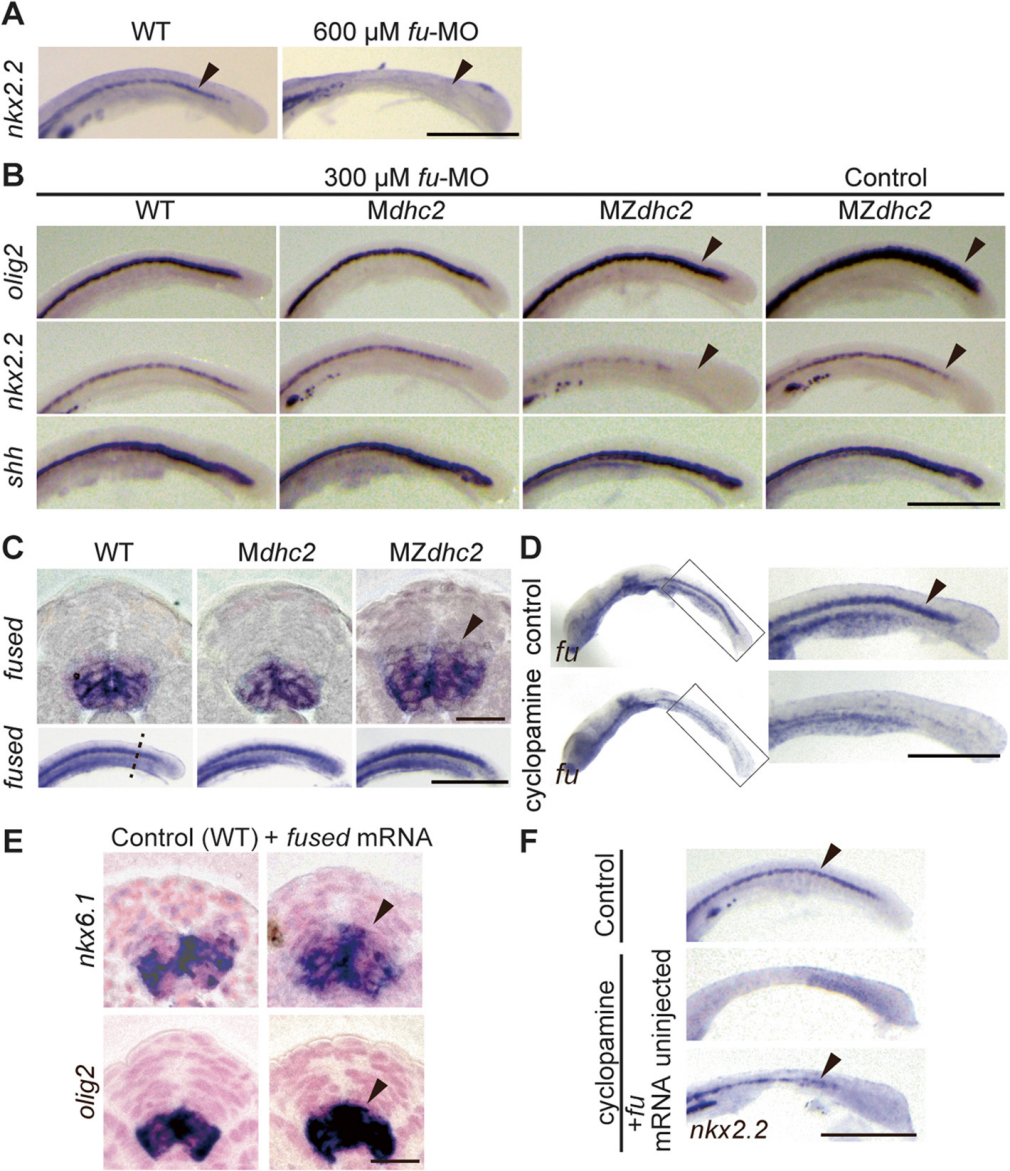
***fused*** **is a Hh target gene in medaka fish. (A-B)** *nkx2.2* expression in 600 μM *fused*-MO injected WT embryos **(A)**, and *shh*, *nkx2.2* and *olig2* expression in 300 μM *fused*-MO injected embryos **(B)**. **(C)** *fu* expression in a cross-sectional view and a lateral view (dashed line indicates section plane). **(D)** *fu* expression in 5 μM cyclopamine-treated embryos. **(E)** *fu* overexpression induced ectopic *nkx6.1* and *olig2* expression (arrowheads). **(F)** The loss of *nkx2.2* expression in 2.5 μM cyclopamine-treated embryos was rescued by overexpression of *fused*. Scale bars: 500 μm in **A**, **B**, **C** (lower panel), **D**, **F**; 20 μm in **C** (upper panel), **E**.

The presence of *nkx2.2* expression is unique in MZ*dhc2* as mouse *Dnchc2* mutants lose *nkx2.2* expression [14,15]. The same tendency was observed in *ift88* mutants that completely lack cilia; only zebrafish mutants maintain the expression of intermediate genes like *olig2* [4,5], implying that the activation of Hh signal is enhanced in fish. To explore a teleost-specific mechanism, we focused on *fused* (*fu*), an intracellular mediator of Hh signaling downstream of Smo in *Drosophila*, which has evolved divergent roles in the vertebrate lineage: one for Hh signaling and the other for ciliary motility. Interestingly, murine *Fu* is dispensable for Hh signaling and specifically participates in the motility of cilia, whereas it is required for both in zebrafish [7,10]. We first tested if *fu* is essential for Hh signaling in medaka by injecting *fu* MO (600 μM) targeted to the splicing site (Additional file 10: Figure S8A) and observed the loss of *nkx2.2* expression (Figure 6A; n = 14/15). Additionally, morphants injected together with *fu* mRNA rescued *nkx2.2* expression (Additional file 10: Figure S8B; n = 14/14) and injection of *fu* mRNA into WT embryos elevated Hh activity as indicated by the expansion of the ventral intermediate genes, *olig2* and *nkx6.1* (Figure 6E, Additional file 10: Figure S8C; n = 9/14, 9/9, respectively). We then knocked down *fu* in MZ*dhc2* mutants to see if the remaining expression of Hh target genes in those mutants also depends on Fu. However, under our experimental conditions, most of the MZ*dhc2* mutants injected with *fu* MO (600 μM) died probably due to a requirement of Fu in earlier development [22], and we therefore reduced the concentration of *fu* MO (300 μM), when injected into MZ*dhc2* mutants. These injected MZ*dhc2* embryos failed to express *nkx2.2* (Figure 6B). Interestingly, the expansion of the ventral intermediate gene, *olig2*, was also rescued (Figure 6B). These results demonstrate that *fu* is indispensable for Hh signaling in wild-type and mutant medaka embryos and its overexpression augments the signal.

*fu* is known to be expressed ubiquitously in zebrafish at early developmental stages [22], but the precise pattern and regulation of *fu* expression during neural tube patterning have not been reported. Our further analysis revealed that *fu* expression is restricted to the ventral part of neural tube where high to low levels of Hh signaling are activated at 16-somite stage in medaka (Figure 6C). Furthermore, *fu* expression was dorsally expanded in MZ*dhc2* neural tubes (Figure 6C), like the ventral intermediate genes. These results suggest that *fu* is a transcriptional target of Hh signaling. To test this possibility, we treated wild-type embryos with 5 μM cyclopamine and observed severe reduction or loss of *fu* expression in cyclopamine-treated embryos (Figure 6D), indicating that *fu* expression is induced by Hh signaling downstream of Smo. We also confirmed that *fu* expression in zebrafish is ventrally restricted in the neural tube and depends on Hh signaling (Additional file 10: Figure S8D).

**Figure 7:**
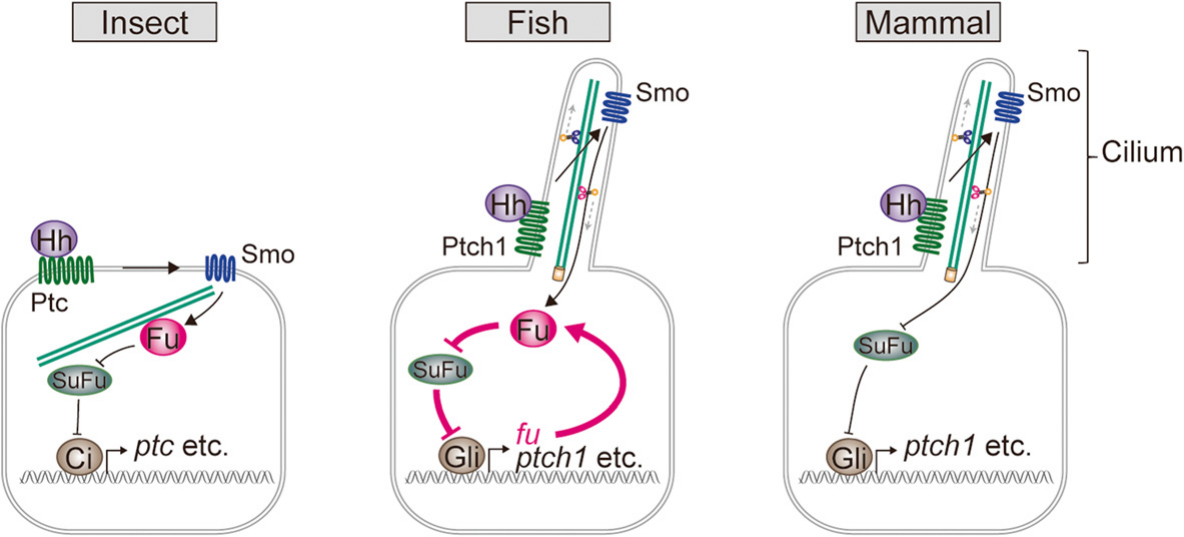
**Proposed model of the distinct features of Hh signal transduction in insect, fish and mammal.** *fu* is expressed in a Hedgehog-dependent fashion and is also one of the components of the Hedgehog pathway in fish. Fused negatively regulates Suppressor of Fused (SuFu), which is a negative regulator of Gli/Ci in Hh signaling. The transcription of *fused* in fish could lead to Hh activation. This positive-feedback loop amplifies Hedgehog pathway in fish downstream of cilia.

We finally asked if Fu, when overexpressed, can restore Hh signaling, when Smo-mediated signaling is compromised. For this, embryos were treated with 2.5 μM cyclopamine (intermediate dose, Figure 5A) together with *fu* mRNA injection. Those injected embryos showed weak but significant up-regulation of *nkx2.2* (n = 12/18) as compared with cyclopamine-treated control embryos (n = 1/14) (Figure 6F), suggesting that Fu augments Hh activity downstream of Smo. Given that Fu is a positive mediator of Hh signal transduction, Fu is likely to form a positive feedback loop downstream of Smo to reinforce Hh signal in teleost target cells (Figure 7).

## Discussion

In the present study, utilizing the medaka mutant with severely shortened cilia, MZ*dhc2*, we demonstrated that shorter cilia mediate less Hh activation in fish. This result suggests that they mediate Hh signaling in a length-dependent manner. We also found that Ptch1 receptor is exclusively localized on the cilium in fish. These are largely consistent with the observation of murine ciliary mutants. Furthermore, the present study has addressed why the expression of low-threshold target genes is expanded in mutant neural tubes and how Hh signal is augmented in fish mutant cells.

### A possible role for cilia in Hh gradient formation

*olig2*-positive wild-type cells in mutant neural tubes were positioned more dorsally than dorsal boundary of *olig2* expression in wild-type neural tubes (Figure 5D). This result, though indirectly, suggest that the gradient profile of Hh ligand dorsally shifts in mutant neural tubes.

Dorsal expansion of Shh ligand was directly observed with *smoothened* mouse mutants [23] and this can be interpreted as a consequence of the reduced amount of Ptch1 receptor, a downstream target of Hh signaling. Indeed it has been proposed that the Shh gradient is regulated by a Shh-induced negative-feedback mechanism in which ligand binding to Ptch1 at the cilia sequesters Hh ligand itself in the intercellular space [18]. It is thus conceivable that in ciliary mutant neural tubes, the reduced amount of Ptch1 on cilia caused a dorsally shifted Hh gradient, and thereby the expression domain of ventral low-threshold target genes is expanded, although further confirmation by direct imaging is required. Consistently, the neural tube in *Dnchc2* mutants also exhibits the expansion of low threshold gene expression [14,15]. Thus in vertebrates, the length of cilia could be one of the factors that affect the Hh gradient in the neural tube. Of course, we still cannot rule out the possibility that wild-type cells, when placed in mutant background, become more sensitive than those in a control background.

### Teleost-specific augmentation of Hh pathway mediated by *fused*

In the present study, we propose that Fu is a key to account for the difference in activation level between mammals and fish in ciliary mutants. Fused is a crucial mediator of Hh signaling in *Drosophila* and zebrafish, but not in mammals [7]. We first confirmed that *fu* was required for Hh signaling in medaka like zebrafish, and next found that the expression of *fu* in neural tube is restricted to the ventral part and induced by Hh signaling in fish. Subsequent analyses demonstrated that Fu forms a positive-feedback loop downstream of Smo (Figure 7); Fu activates the Hh pathway which then leads to the up-regulation of Fu. The positive feedback centered by Fu could augment Hh signal in ciliary mutant cells with lower input of Smo-mediated signaling. This could thus explain why the phenotype of fish ciliary mutants are milder than that of mammalian counterparts. Of course, Fu may not be a mere component that differentiates the ciliary dependency in the two vertebrate models. Indeed, in zebrafish, low levels of Hh activation mediated by Gli1 are known to occur in a Hh-independent manner and its mechanism remain elusive [5].

What is the biological and evolutionarily significance of the positive-feedback mechanism in Hh signaling. A hint could be found in the speed and mode of neurulation in fish. According to the recent report by Xiong et al. [24], specification of neural cell types in zebrafish begins earlier and proceeds faster under the noisy conditions of cell movements in the formation of the neural keel. Whereas in other vertebrates, such as chick and mice, neurulation proceeds gradually and steadily in an epithelialized cell sheet, following an established Shh gradient. A rapid and amplified response to Shh in target cells would thus be necessary in fish neurulation. Xiong et al. also showed that specified neural progenitors sort to form sharply bordered domains from mixed progenitor populations. However, this apparently contradicts our transplantation result showing ectopic expression of a specific marker in wild-type donors (Figure 5D), suggesting that multiple strategies, including sorting and position-dependent determination, are employed to achieve a robust pattern. Recently, the presence of cilium-mediated signaling was reported in the olfactory epithelium of *Drosophila* [24], suggesting the evolutionarily ancient origin of this mechanism. Thus, further analysis of Hh signaling in diverse species and tissues will provide greater insight into the evolution of this crucial signaling pathway.

## Conclusion

The present study strengthens the idea of a conserved role of primary cilia in Hh-signal transduction in vertebrates, but also uncovered a teleost-specific augmentation mechanism mediated by Fu. The fish-specific augmentation can serve as the mechanism that accounts for the lower cilia-dependency for Hh signaling in fish and gives novel insight into the evolution of Hh signaling.

## Methods

### Fish strains

All studies of medaka (*Oryzias latipes*) were carried out using d-rR strain of a closed colony. And zebrafish (*Danio rerio*) were Riken wild-type (RW). All experimental procedures and animal care were carried out according to the animal ethics committee of the University of Tokyo.

### Whole mount *in situ* hybridization

*In situ* hybridization analyses were performed as previously described [25]. The cDNAs used as the templates for the probes were described in Additional file 11: Table S1.

### Histology

For histological analysis, fixed embryos were embedded in Technovit 7100 (Heraeus Kulzer). Scanning electron-microscope observations were performed as previously described [26].

### Immunofluorescence

Whole-mount immunostaining was performed as described previously [27]. The antibodies used were as follows: Polyclonal anti-medaka Ptch1 [amino acids 169–405; Additional file 7: Figure S6B] antibody were raised by immunization of rabbits with bacterial-expressed His-tagged truncated proteins and the antibody was affinity-purified as described [28]; acetylated α-tubulin (Sigma); γ-tubulin (Sigma).

### Chemical treatment

For cyclopamine (Enzo Life Sciences) treatment, dechorionated embryos were incubated from 30-50% epiboly stage onward.

## Additional files

Additional file 1: Figure S1. The medaka *aA90*/*dhc2* lacks essential domains of the *dhc2* gene. **(A)** Positional cloning of the *aA90* mutation in linkage group (LG) 13. The number of recombinants at each marker is shown. **(B)** Genotyping for *aA90*/*dhc2*. A small segment of caudal fin was excised and genomic DNA was extracted with 50 μl of DNA extraction buffer [10 mM Tris pH 8.0, 50 mM KCl, 0.3% Tween20, 0.3% NP40 and 1 mg/ml proteinase K (Invitrogen)]. 2 μl lysate was used for PCR detection using the primers described in arrows of Figure S1A (Additional file 11: Table S1). In *aA90*/*dhc2* mutants, the aberrant products detected by genomic PCR give rise to presumptive truncated forms of Dhc2 that lack HC-HC & HC-IC interaction domain and AAA ATPase domain (Top and middle panels; Figure S1C). **(C)** Schematic diagrams of Dhc2 protein expressed in wild-type and the deleted region in mutants. HC, heavy chain; IC, intermediate chain; MT, microtubule; a.a., amino acids. **(D)** Expression analysis of *dhc2* at 6 dpf by RT-PCR using primers described in Figure S1C (arrows) and Additional file 11: Table S1. The *dhc2* expression was diminished in homozygous *aA90*/*dhc2* mutants. Additional file 2: Figure S2. Medaka MZ*dhc2* mutants have shortened cilia in Kupffer’s vesicle. Cilia were visualized by staining with anti-acetylated α-tubulin antibody (green) and basal bodies were visualized by staining with anti-γ-tubulin antibody (magenta). Cilia are shortened in the Kupffer’s vesicle in MZ*dhc2* mutants, compared with that in control embryos. Scale bar: 5 μm. Additional file 3: Figure S3. Neural and somite patterning in MZ*dhc2* mutant embryos. **(A-J)** Expression of neural tube markers in MZ*dhc2* mutant embryos. Wild type and M*dhc2* control medaka embryos and MZ*dhc2* mutants were stained at 16-somite stage for the expression of *dbx2* (A), *dbx1* (B), *pax3* (C), *pax6* (D), *nkx6.2* (E), *nkx6.1* (F), *olig2* (G), *nkx2.2* (H), *foxa2* (I), *shh* (J) in a lateral view. MZ*dhc2* mutants show *shh*, *foxa2* and *nkx2.2* expression (H, I, J), dorsally expanded expression of *olig2*, *nkx6.1* and *nkx6.2* (E, F, G; arrowheads), and retracted expression of *dbx* genes, *pax6* and *pax3* (A, B, C, D; arrowheads) in the neural tube. Cross-sectional views at the dashed line in A were depicted in Figure 3C. **(K)** Somite patterning in MZ*dhc2* embryos. Adaxial cells (*engrailed1*-positive cells) were significantly decreased in MZ*dhc2* as compared with control embryos. **(L)** *ptch1* expression in MZ*dhc2* is nearly identical to that in WT. **(M)** dnPKA mRNA injected MZ*dhc2* exhibited ectopic *nkx2.2* expression (n = 19/24, arrowhead), consistent with dnPKA mRNA injected-WT (n = 22/24, arrowhead) and M*dhc2* embryos (n = 17/20, arrowhead), compared with Control (WT) embryos. Scale bars: 500 μm in L and M. Additional file 4: Figure S4.
*nkx2.2* and *olig2* expression at three AP-axis levels. **(A)** *nkx2.2* and *olig2* expression at three different AP-axis levels of 16-somite stage embryos. **(B)** *olig2* expression in WT for indicating the position of Anterior (A), Middle (M) and Posterior (P) level, depicted in A and C. **(C)** Measurement of the dorsal boundary of *nkx2.2* and *olig2* expression at relative distances (percentage (%) of the neural tube) from the floor plate in WT and MZ*dhc2* of 16-somite stage (n ≥ 3 embryos; mean ± SD). For the representation of the dorsal boundary of *nkx2.2* and *olig2* expression in same graph, blue shade is for *nkx2.2* and red shade is for *olig2* expression. The *olig2* boundary in mutant embryos is significantly different from WT counterparts (p values from Student’s t test: Anterior, p < 0.05; Middle, p < 0.0005; Posterior, p < 0.005). Additional file 5: Figure S5. Dose-dependent effects of cyclopamine treatment on the expression of Hh target genes. **(A)** *foxa2*, *nkx2.2* and *olig2* expression in embryos treated with DMSO, 2.5 μM, 5 μM cyclopamine. **(B)** *foxa2* expression is absent in the lateral FP and only detectable in the medial FP in embryos treated with 5 μM cyclopamine, when compared to the DMSO control. Additional file 6: Table S3. Number of samples to examine Hh activity with the graded series of cyclopamine treatment depicted in Figure 5A. Additional file 7: Figure S6. Anti-Ptch1 antibody specifically recognizes medaka Ptch1. **(A)** Phylogenetic trees showing the relationship between Patched proteins across vertebrates based on neighbor-joining method and maximum likelihood estimation. o, *Oryzias latipes* (medaka); g, *Gallus gallus*; h, *Homo sapiens*; m, *Mus musculus*; x, *Xenopus tropicalis*; d, *Danio rerio*). All sequences are obtained from Ensembl Web site and the accession numbers are listed on Additional file 8: Table S2. **(B)** His-tagged N-terminal (169–405; Ptch1- His) polypeptides of medaka Ptch1 (orange lined) were expressed in *E. coli* Rosetta (DE3) competent cells using pET24a (Novagen) and purified with Profinity™ IMAC Ni-charged resin (Bio-Rad) under denaturing conditions and dialyzed against PBS. The polypeptides were used for immunization of rabbits. **(C)** Ptch1 were visualized by staining with anti-medaka Ptch1 antibody (green) and cilia were visualized with anti-acetylated α-tubulin antibody (red). Ptch1 morpholino antisense oligo for splicing blocking (intron 5 and exon 6) (5′-CCCCTACCTCTGTAAAGTTAATTAC-3′) injected embryos had no Ptch1 positive signals. **(D)** Injection of *ptch1*-morpholino induced ectopic Hh-dependent muscle pioneer (Eng + cells, lateral view, arrowheads), visualized by staining with anti-Engrailed antibody (4D9). **(E)** Ptch1-myc (magenta) was specifically localized to cilia in neural tube and the signals are well merged with anti-Ptch1 antibody signals (green) in a cross-sectional view at 16-somite stage. Additional file 8: Table S2. Accession numbers used to create the phylogenetic trees depicted in Additional file 7: Figure S6A. Additional file 9: Figure S7. Optic cup and lens formation were significantly defected in MZ*dhc2*, as compared with WT, Z*dhc2* and M*dhc2*. Dorsal views of the eye in WT, Z*dhc2*, M*dhc2* and MZ*dhc2* at 16-somite stage. Arrows indicate optic cup and arrow heads indicate lens. Scale bar: 100 μm. Additional file 10: Figure S8.
*fu* knockdown, overexpression in medaka, and *fu* expression pattern in Zebrafish. **(A)** Knockdown of *fu* was performed using the morpholino-oligonucleotide (MO) for splice blocking (5′-CAACCACCTTATTGACGACAAAACA-3′). Diagram of altered *fu* splicing in morphants of *fu*-i1e2 inserts intron 1 (+In. 1), resulting in an out-of-frame truncation of the *fu* protein, and splices exon 2 to a cryptic acceptor in exon 3 (− Ex. 2), causing an out-frame mutation of *fu*. The effect of the splice-blocking MO was verified by RT-PCR from 20 embryos total RNA (16-somite stage). Primers for checking the effect of MO were indicated in A (arrows). MO caused splice-blocking effectively. **(B)** *fu* mRNA injection rescued *nkx2.2* expression in *fu* morpholino injected embryos. **(C)** *fu* overexpression induced ectopic *nkx6.1* and *olig2* expression **(D)** *fu* expressed in Hedgehog-dependent fashion also in zebrafish. The embryos treated with cyclopamine did not express *fused* or *nkx2.2a*. Scale bar: 500 μm in lateral view in B, C, D; 20 μm in cross-section in D. Additional file 11: Table S1. Primers used in this study.

## Abbreviations

*dhc2*: *cytoplasmic dynein heavy chain 2*
dpf: Days post-fertilization
ENU: N-ethyl-N-nitrosourea
*fu*: *fused*
GFP: Green fluorescent protein
HC: Heavy chain
Hh: Hedgehog
IC: Intermediate chain
IFT: Intrafllagelar transport
KV: Kupffer’s vesicle
LNT: Lateral neural tube
M*dhc2*: Maternal mutant of *dhc2*
MO: Morpholino antisense oligonucleotide
MZ*dhc2*: Maternal-zygotic mutant of *dhc2*
ORF: Open reading frame
Ptch1: Patched 1
Shh: Sonic hedgehog
Smo: Smoothened
VM: Ventral midline
WT: Wild type
Z*dhc2*: Zygotic mutant of *dhc2*
