# Supplementary figures and images for: The medaka dhc2 mutant reveals conserved and distinct mechanisms of Hedgehog signaling in teleosts
Source: BMC Dev Biol. 2015 Feb 3;15:9. doi: 10.1186/s12861-015-0057-x (PMC4320493; doi:10.1186/s12861-015-0057-x)

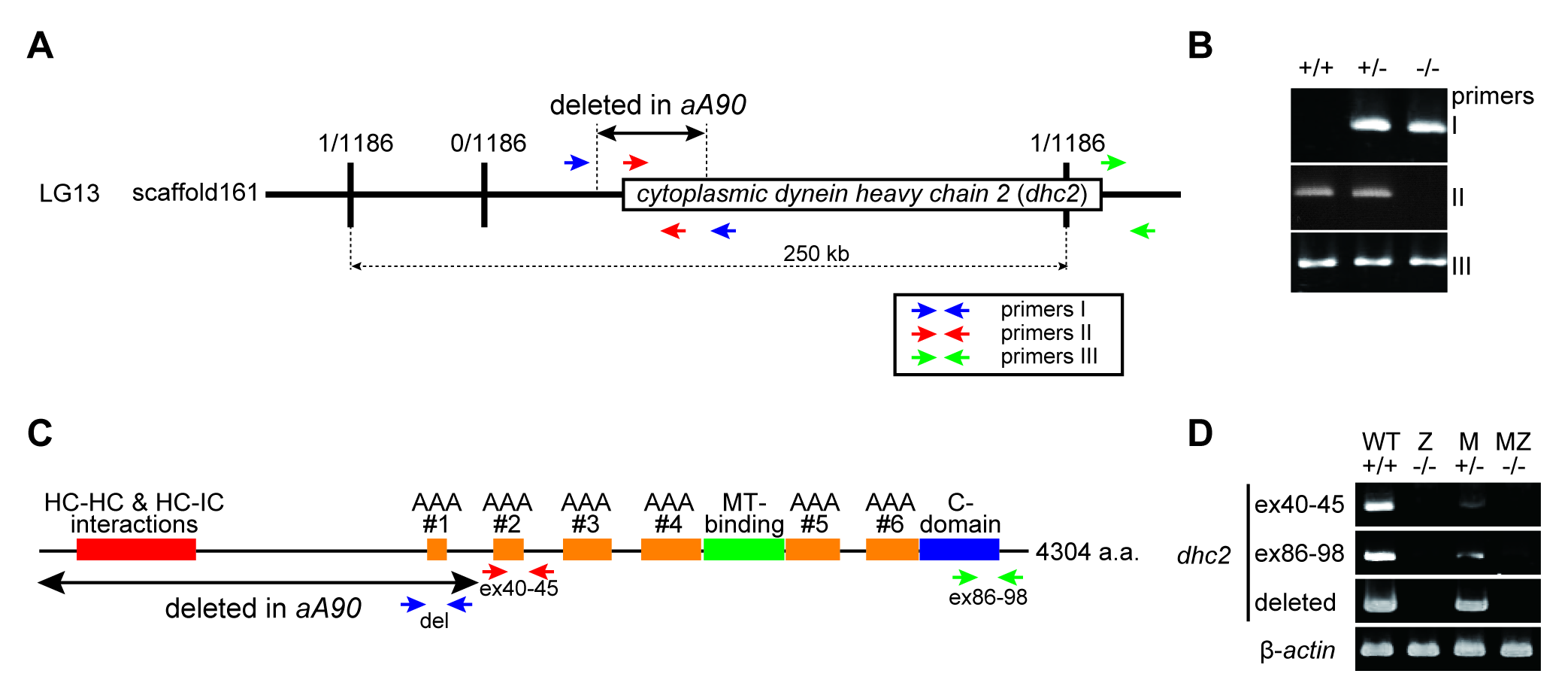

Supplement: Additional file 1: Figure S1. — The medaka aA90/dhc2 lacks essential domains of the dhc2 gene. (A) Positional cloning of the aA90 mutation in linkage group (LG) 13. The number of recombinants at each marker is shown. (B) Genotyping for aA90/dhc2. A small segment of caudal fin was excised and genomic DNA was extracted with 50 μl of DNA extraction buffer [10 mM Tris pH 8.0, 50 mM KCl, 0.3% Tween20, 0.3% NP40 and 1 mg/ml proteinase K (Invitrogen)]. 2 μl lysate was used for PCR detection using the primers described in arrows of Figure S1A (Additional file 11: Table S1). In aA90/dhc2 mutants, the aberrant products detected by genomic PCR give rise to presumptive truncated forms of Dhc2 that lack HC-HC & HC-IC interaction domain and AAA ATPase domain (Top and middle panels; Figure S1C). (C) Schematic diagrams of Dhc2 protein expressed in wild-type and the deleted region in mutants. HC, heavy chain; IC, intermediate chain; MT, microtubule; a.a., amino acids. (D) Expression analysis of dhc2 at 6 dpf by RT-PCR using primers described in Figure S1C (arrows) and Additional file 11: Table S1. The dhc2 expression was diminished in homozygous aA90/dhc2 mutants. [file 12861_2015_57_MOESM1_ESM.tiff]

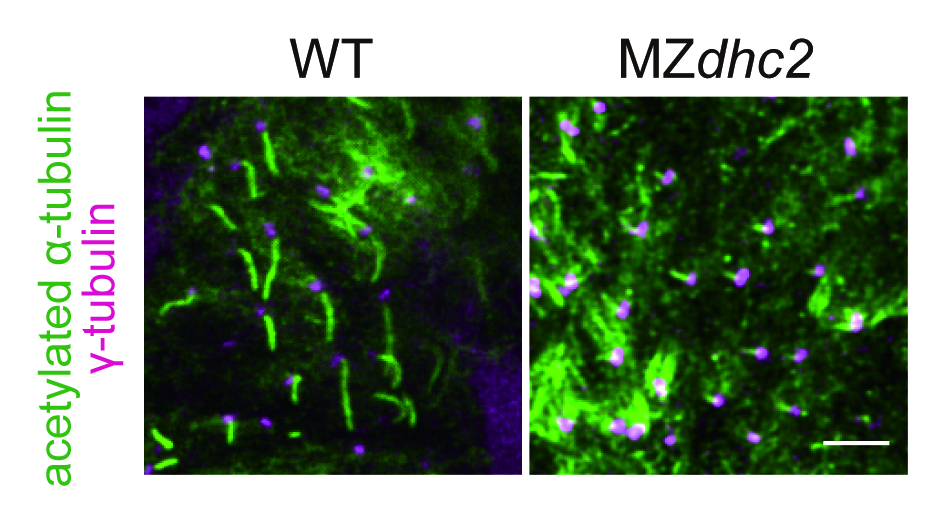

Supplement: Additional file 2: Figure S2. — Medaka MZdhc2 mutants have shortened cilia in Kupffer’s vesicle. Cilia were visualized by staining with anti-acetylated α-tubulin antibody (green) and basal bodies were visualized by staining with anti-γ-tubulin antibody (magenta). Cilia are shortened in the Kupffer’s vesicle in MZdhc2 mutants, compared with that in control embryos. Scale bar: 5 μm. [file 12861_2015_57_MOESM2_ESM.tiff]

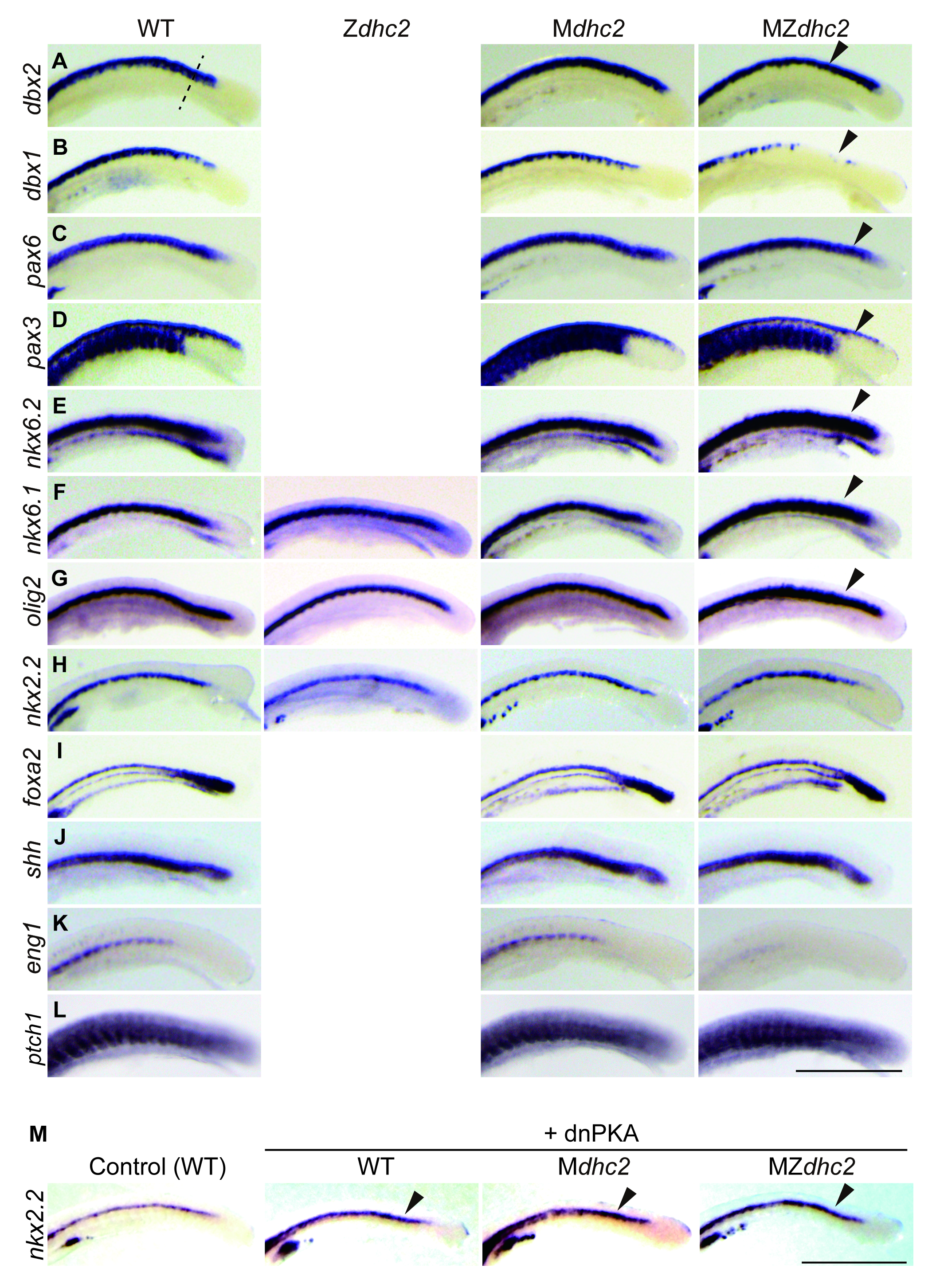

Supplement: Additional file 3: Figure S3. — Neural and somite patterning in MZdhc2 mutant embryos. (A-J) Expression of neural tube markers in MZdhc2 mutant embryos. Wild type and Mdhc2 control medaka embryos and MZdhc2 mutants were stained at 16-somite stage for the expression of dbx2 (A), dbx1 (B), pax3 (C), pax6 (D), nkx6.2 (E), nkx6.1 (F), olig2 (G), nkx2.2 (H), foxa2 (I), shh (J) in a lateral view. MZdhc2 mutants show shh, foxa2 and nkx2.2 expression (H, I, J), dorsally expanded expression of olig2, nkx6.1 and nkx6.2 (E, F, G; arrowheads), and retracted expression of dbx genes, pax6 and pax3 (A, B, C, D; arrowheads) in the neural tube. Cross-sectional views at the dashed line in A were depicted in Figure 3C. (K) Somite patterning in MZdhc2 embryos. Adaxial cells (engrailed1-positive cells) were significantly decreased in MZdhc2 as compared with control embryos. (L) ptch1 expression in MZdhc2 is nearly identical to that in WT. (M) dnPKA mRNA injected MZdhc2 exhibited ectopic nkx2.2 expression (n = 19/24, arrowhead), consistent with dnPKA mRNA injected-WT (n = 22/24, arrowhead) and Mdhc2 embryos (n = 17/20, arrowhead), compared with Control (WT) embryos. Scale bars: 500 μm in L and M. [file 12861_2015_57_MOESM3_ESM.tiff]

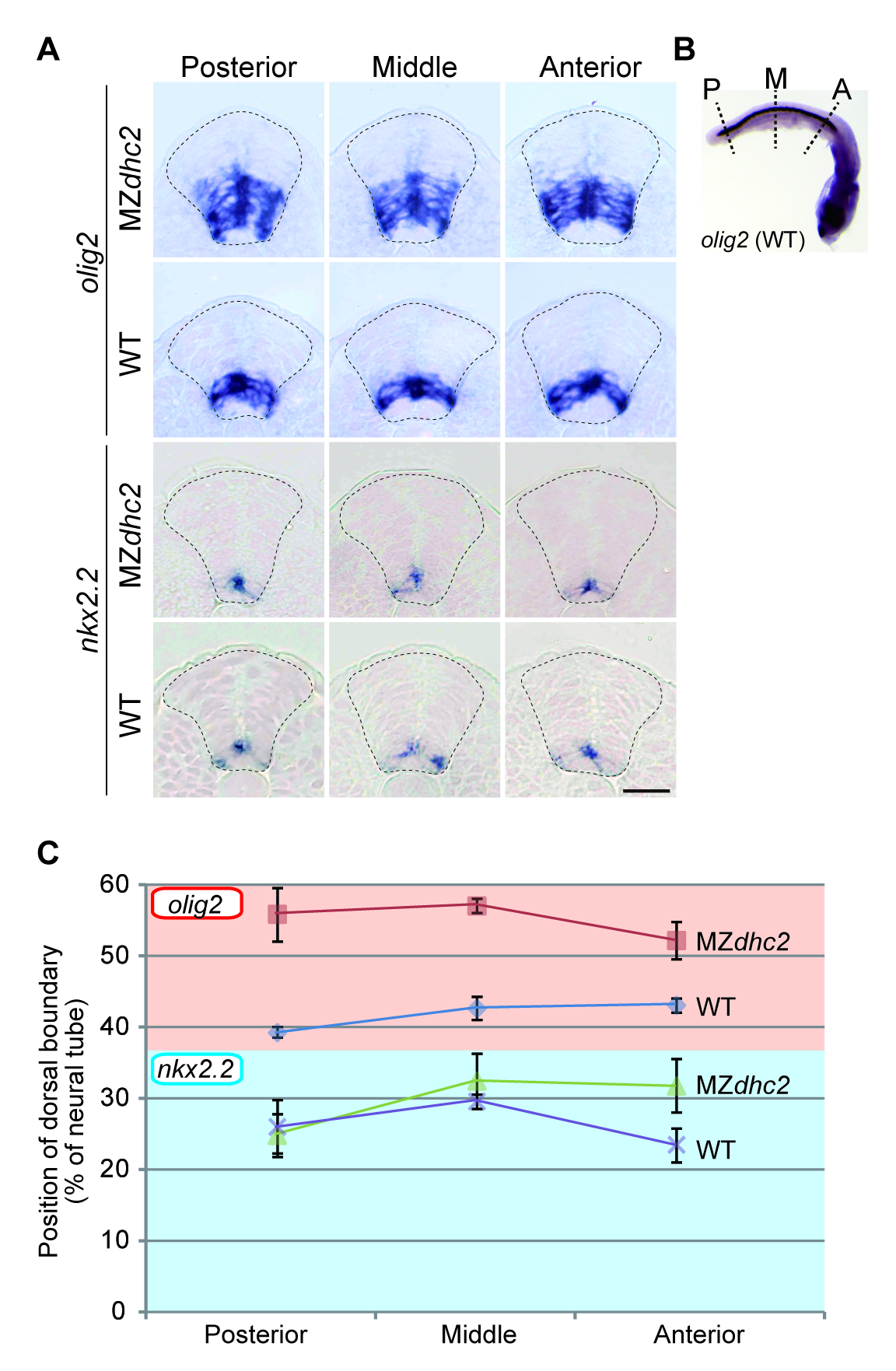

Supplement: Additional file 4: Figure S4. — nkx2.2 and olig2 expression at three AP-axis levels. (A) nkx2.2 and olig2 expression at three different AP-axis levels of 16-somite stage embryos. (B) olig2 expression in WT for indicating the position of Anterior (A), Middle (M) and Posterior (P) level, depicted in A and C. (C) Measurement of the dorsal boundary of nkx2.2 and olig2 expression at relative distances (percentage (%) of the neural tube) from the floor plate in WT and MZdhc2 of 16-somite stage (n ≥ 3 embryos; mean ± SD). For the representation of the dorsal boundary of nkx2.2 and olig2 expression in same graph, blue shade is for nkx2.2 and red shade is for olig2 expression. The olig2 boundary in mutant embryos is significantly different from WT counterparts (p values from Student’s t test: Anterior, p < 0.05; Middle, p < 0.0005; Posterior, p < 0.005). [file 12861_2015_57_MOESM4_ESM.tiff]

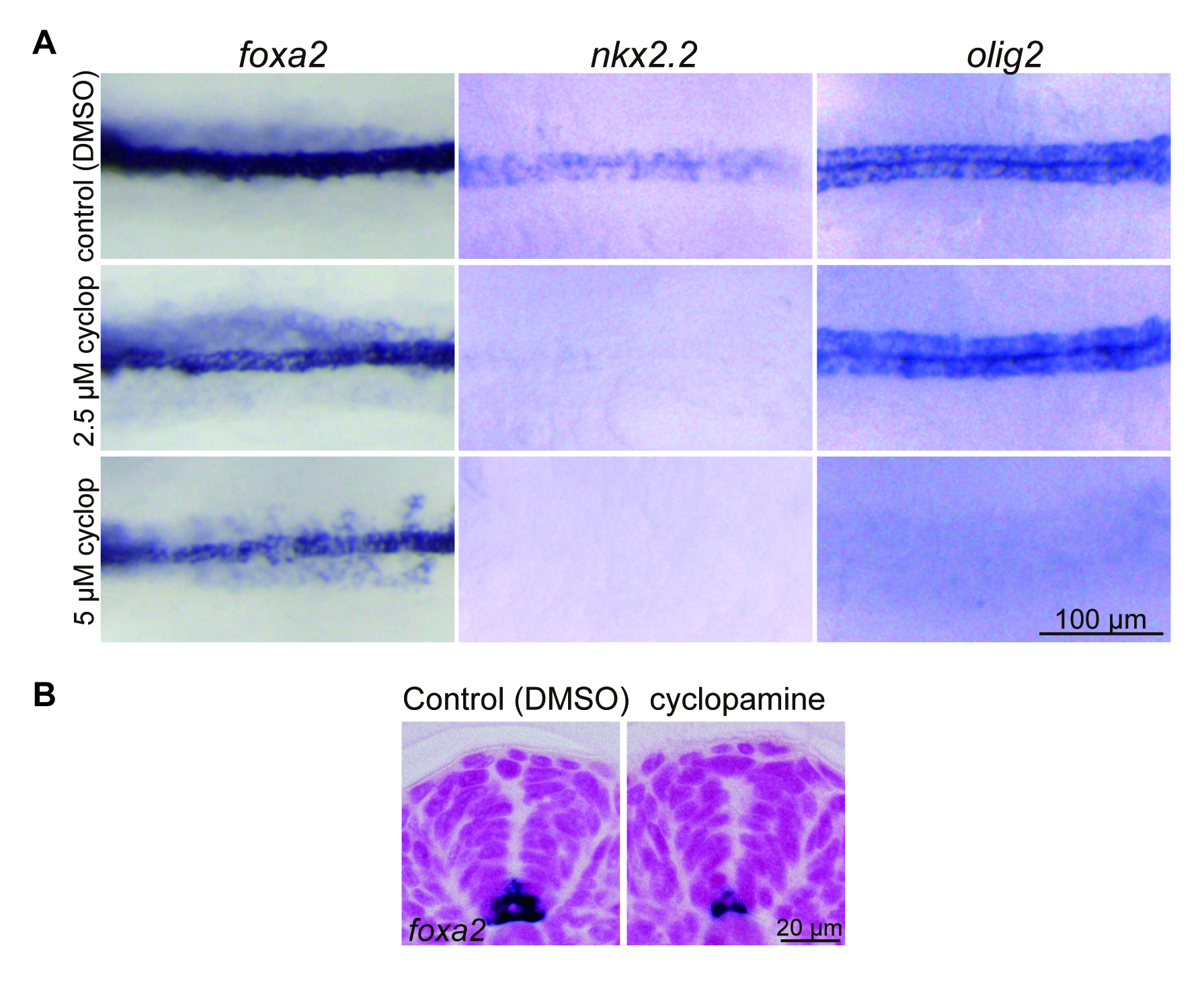

Supplement: Additional file 5: Figure S5. — Dose-dependent effects of cyclopamine treatment on the expression of Hh target genes. (A) foxa2, nkx2.2 and olig2 expression in embryos treated with DMSO, 2.5 μM, 5 μM cyclopamine. (B) foxa2 expression is absent in the lateral FP and only detectable in the medial FP in embryos treated with 5 μM cyclopamine, when compared to the DMSO control. [file 12861_2015_57_MOESM5_ESM.tiff]

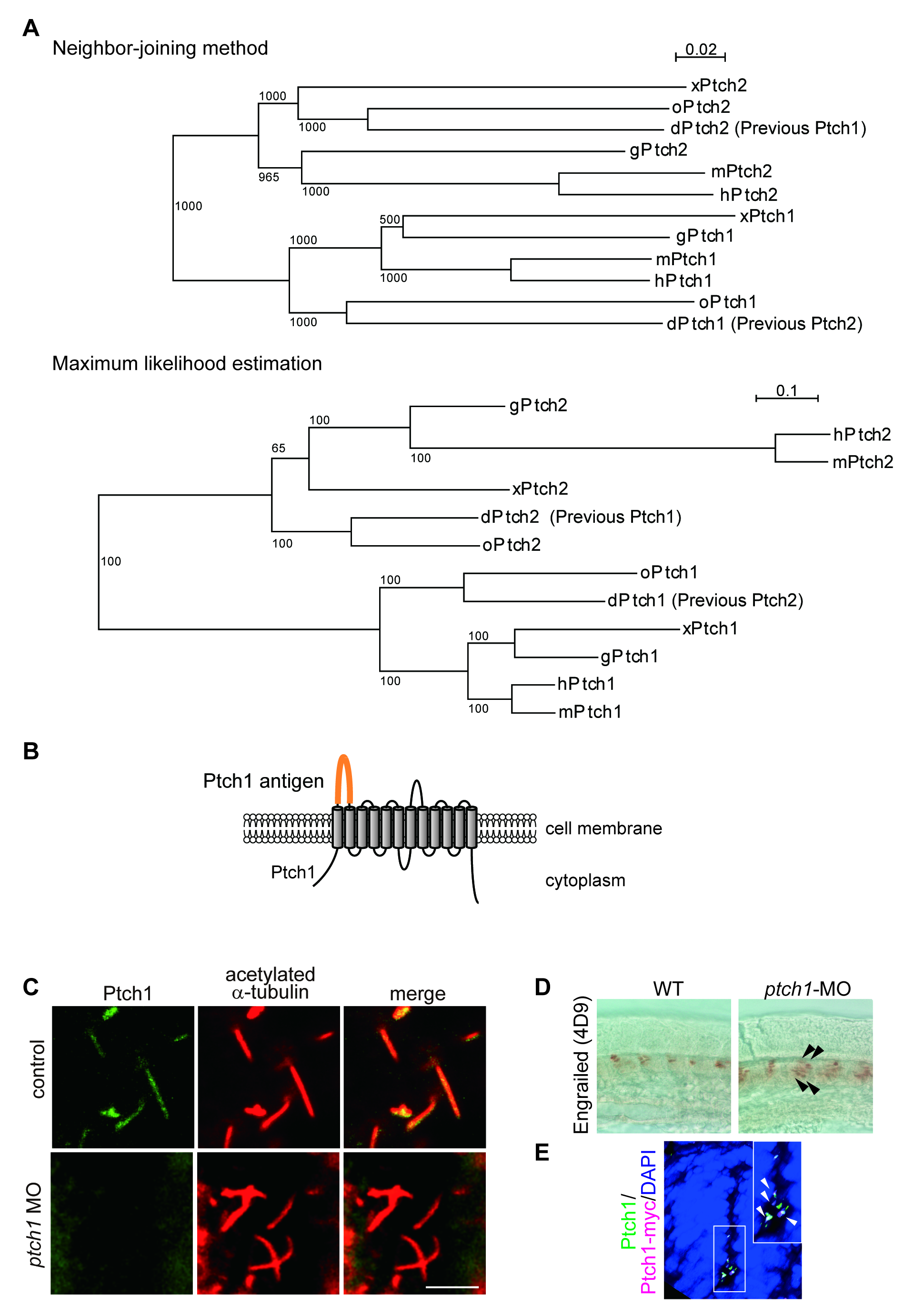

Supplement: Additional file 7: Figure S6. — Anti-Ptch1 antibody specifically recognizes medaka Ptch1. (A) Phylogenetic trees showing the relationship between Patched proteins across vertebrates based on neighbor-joining method and maximum likelihood estimation. o, Oryzias latipes (medaka); g, Gallus gallus; h, Homo sapiens; m, Mus musculus; x, Xenopus tropicalis; d, Danio rerio). All sequences are obtained from Ensembl Web site and the accession numbers are listed on Additional file 8: Table S2. (B) His-tagged N-terminal (169–405; Ptch1- His) polypeptides of medaka Ptch1 (orange lined) were expressed in E. coli Rosetta (DE3) competent cells using pET24a (Novagen) and purified with Profinity™ IMAC Ni-charged resin (Bio-Rad) under denaturing conditions and dialyzed against PBS. The polypeptides were used for immunization of rabbits. (C) Ptch1 were visualized by staining with anti-medaka Ptch1 antibody (green) and cilia were visualized with anti-acetylated α-tubulin antibody (red). Ptch1 morpholino antisense oligo for splicing blocking (intron 5 and exon 6) (5′-CCCCTACCTCTGTAAAGTTAATTAC-3′) injected embryos had no Ptch1 positive signals. (D) Injection of ptch1-morpholino induced ectopic Hh-dependent muscle pioneer (Eng + cells, lateral view, arrowheads), visualized by staining with anti-Engrailed antibody (4D9). (E) Ptch1-myc (magenta) was specifically localized to cilia in neural tube and the signals are well merged with anti-Ptch1 antibody signals (green) in a cross-sectional view at 16-somite stage. [file 12861_2015_57_MOESM7_ESM.tiff]

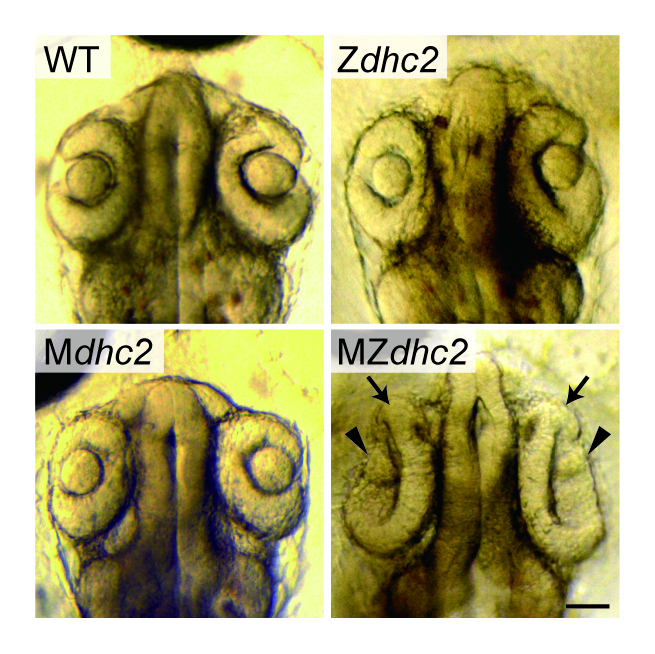

Supplement: Additional file 9: Figure S7. — Optic cup and lens formation were significantly defected in MZdhc2, as compared with WT, Zdhc2 and Mdhc2. Dorsal views of the eye in WT, Zdhc2, Mdhc2 and MZdhc2 at 16-somite stage. Arrows indicate optic cup and arrow heads indicate lens. Scale bar: 100 μm. [file 12861_2015_57_MOESM9_ESM.tiff]

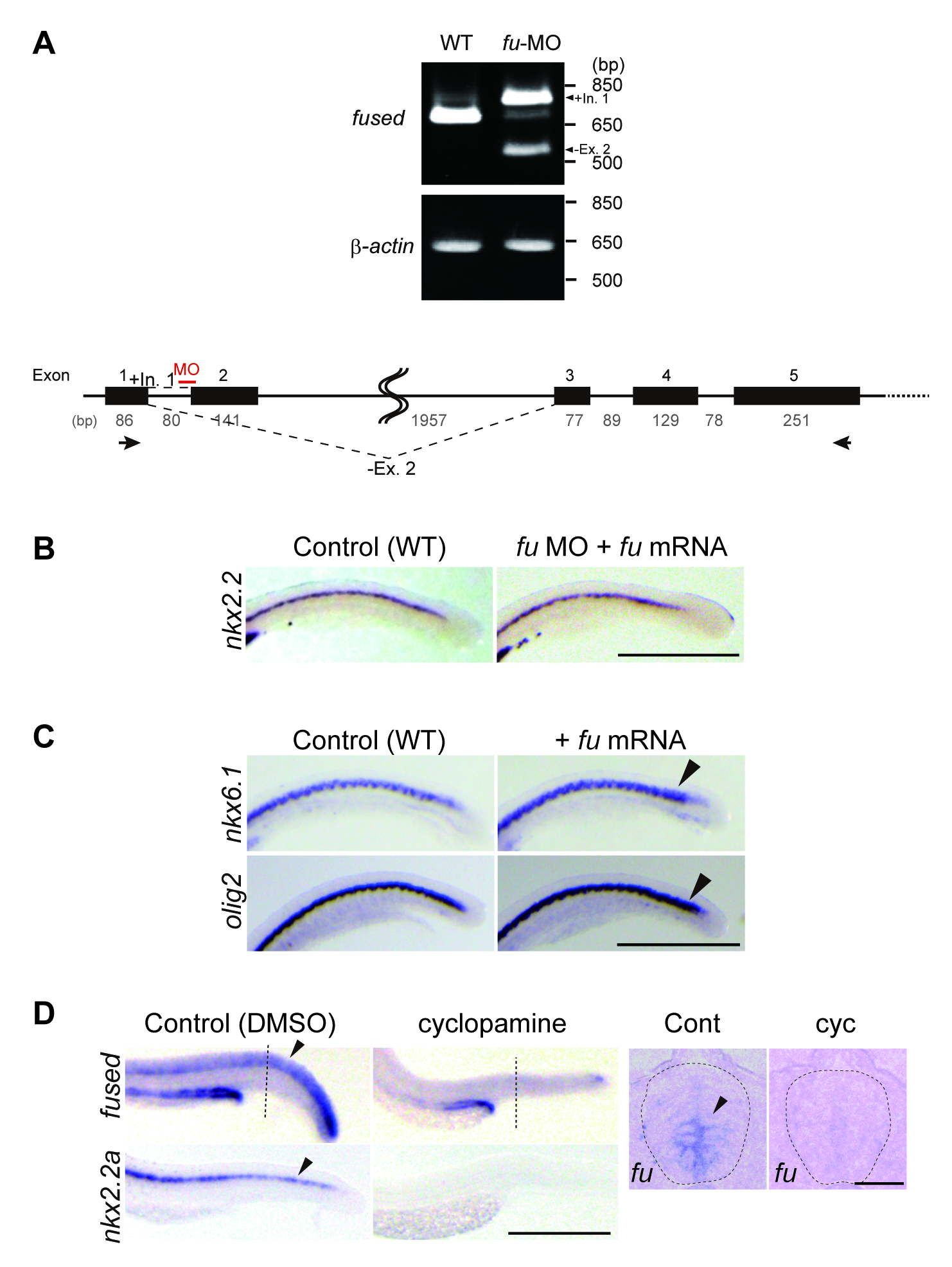

Supplement: Additional file 10: Figure S8. — fu knockdown, overexpression in medaka, and fu expression pattern in Zebrafish. (A) Knockdown of fu was performed using the morpholino-oligonucleotide (MO) for splice blocking (5′-CAACCACCTTATTGACGACAAAACA-3′). Diagram of altered fu splicing in morphants of fu-i1e2 inserts intron 1 (+In. 1), resulting in an out-of-frame truncation of the fu protein, and splices exon 2 to a cryptic acceptor in exon 3 (− Ex. 2), causing an out-frame mutation of fu. The effect of the splice-blocking MO was verified by RT-PCR from 20 embryos total RNA (16-somite stage). Primers for checking the effect of MO were indicated in A (arrows). MO caused splice-blocking effectively. (B) fu mRNA injection rescued nkx2.2 expression in fu morpholino injected embryos. (C) fu overexpression induced ectopic nkx6.1 and olig2 expression (D) fu expressed in Hedgehog-dependent fashion also in zebrafish. The embryos treated with cyclopamine did not express fused or nkx2.2a. Scale bar: 500 μm in lateral view in B, C, D; 20 μm in cross-section in D. [file 12861_2015_57_MOESM10_ESM.tiff]
