# Supplementary material for: The medaka dhc2 mutant reveals conserved and distinct mechanisms of Hedgehog signaling in teleosts
Source: BMC Dev Biol. 2015 Feb 3;15:9. doi: 10.1186/s12861-015-0057-x (PMC4320493; doi:10.1186/s12861-015-0057-x)
Supplement: Additional file 6: Table S3. — Number of samples to examine Hh activity with the graded series of cyclopamine treatment depicted in Figure 5A. [file 12861_2015_57_MOESM6_ESM.docx]

Table S3. Number of samples to examine Hh activity with the graded series of cyclopamine treatment depicted in Fig. 4A.

| cyclopamine (nM) | 250 | 500 | 1000 | 2500 | 5000 |
| --- | --- | --- | --- | --- | --- |
| Wild type | 20 | 23 | 26 | 18 | 17 |
| M*dhc2* | 18 | 19 | 20 | 20 | 18 |
| MZ*dhc2* | 20 | 26 | 19 | 18 | 16 |
