# Supplementary material for: The medaka dhc2 mutant reveals conserved and distinct mechanisms of Hedgehog signaling in teleosts
Source: BMC Dev Biol. 2015 Feb 3;15:9. doi: 10.1186/s12861-015-0057-x (PMC4320493; doi:10.1186/s12861-015-0057-x)
Supplement: Additional file 8: Table S2. — Accession numbers used to create the phylogenetic trees depicted in Additional file 7: Figure S6A. [file 12861_2015_57_MOESM8_ESM.docx]

Table S2. Accession numbers used to create the phylogenetic trees depicted in Fig. S6A.

|  | Ptch1 | Ptch2 |
| --- | --- | --- |
| *Oryzias latipes* | ENSORLG00000004345 | ENSORLG00000016137 |
| *Gallus gallus* | ENSGALG00000012620 | ENSGALG00000010133 |
| *Homo sapiens* | ENSG00000185920 | ENSG00000117425 |
| *Mus musculus* | ENSMUSG00000021466 | ENSMUSG00000028681 |
| *Xenopus tropicalis* | ENSXETG00000014834 | ENSXETG00000018892 |
| *Danio rerio* | ENSDARG00000016404 | ENSDARG00000055026 |
