# Supplementary material for: The medaka dhc2 mutant reveals conserved and distinct mechanisms of Hedgehog signaling in teleosts
Source: BMC Dev Biol. 2015 Feb 3;15:9. doi: 10.1186/s12861-015-0057-x (PMC4320493; doi:10.1186/s12861-015-0057-x)
Supplement: Additional file 11: Table S1. — Primers used in this study. [file 12861_2015_57_MOESM11_ESM.docx]

Additional file 9: Table S1. Primers used in this study

|  | | |  |
| --- | --- | --- | --- |
| Genotype | | Forward (5' to 3') | Reverse (5' to 3') |
| primers I | | CCCGCTGAGTTTAGAGACTATTG | GTGGGAAGTGTACACCTTCATAAT |
| primers II | | TTCTATGGGTGATGCCACTTTC | GGAAATCTGATACAACCCCAGC |
| primers III | | CTCAAAGTGAGCTTTTGGCTCAAGTATT | ACTGTAGAAGATGGGACACGAAGAAAAG |
|  | |  |  |
| Probe* | | Forward (5' to 3') | Reverse (5' to 3') |
| *nkx2.2* | | TCGTTGACCAACACAAAGACGG | CCAAGTCCTGAGCTTTAAGAGTGTG |
| *olig2* | | ATACAAGTCGTGTGTCAAGCAGACC | TGAGAAGTCCGTGATGGGGTC |
| *fused* | | TTCAGTAAAAACGCGTGAGC | AACACGTTTGTGTCCGACAG |
| *nkx6.1* | | TCTTCTGGCCGGGAGTCATG | AAGTGCTTTACATGAAGCTGCG |
| *nkx6.2* | | ATGGAAGCTAACCGGCAGAG | CACTTGGTCCTCCGGTTCTG |
| *pax3* | | CAGGAGGTTTACCAAGAATGATG | AAGACTGAGTACTGGGCAGAGTG |
| *dbx1* | | AAGAAGCGGTTCCTGATTTCTC | CTCATTCTTTCTCCTCCCAACTC |
| *dbx2* | | CTCCTGCTCTGCCAGGTTTTG | CACTGGTGTGATTGTGTGACAG |
| *eng1* | | AACCACCAACTTTTTCATCGAC | ATCTGGGACTCGTTCAGGTG |
| zebrafish *nkx2.2a* | | GCACTCCTTACTTTCATTTGG | CGTATAACACGAAGGACAAAAG |
| zebrafish *fused* | | GGAGAAAACGGTCTAAGTTATG | ATCAGAACTCCATCTGCAAC |
| **shh* (AB007129) and *foxa2* (AB001572) were kindly provided by Dr. K. Araki; *pax6* were by Dr. A. Kawakami. | | | |
|  |  | |  |
| RT-PCR | Forward (5' to 3') | | Reverse (5' to 3') |
| *fused* | ATGAATTCCTATCACGTCTTG | | ATGCAGTTATCACTCATTGTGTC |
| β-*actin* | GATGAAGCCCAGAGCAAGAG | | AGGAAGGAAGGCTGGAAGAG |
| *dhc2* (ex40-45) | GTGCAAGCACTGAGGCTC | | CACTAGACTAGTTTCCACCACAAAG |
| *dhc2* (ex86-98) | CTTTGTCCACGGCCTGTTC | | CTGTTTGAGAAAAAGAGCAGCTC |
| *dhc2* (del) | GTTGAGGTGTGGTTAGGAGAGC | | TGGTTTGCTCATGGCTACG |
